# Specificity and Application of the Lantibiotic Protease NisP

**DOI:** 10.3389/fmicb.2018.00160

**Published:** 2018-02-09

**Authors:** Manuel Montalbán-López, Jingjing Deng, Auke J. van Heel, Oscar P. Kuipers

**Affiliations:** Department Molecular Genetics, University of Groningen, Groningen, Netherlands

**Keywords:** lantibiotic, nisin, bacteriocin, *Lactococcus lactis*, NisP, leader peptidase, subtilisin-like protease

## Abstract

Lantibiotics are ribosomally produced and posttranslationally modified peptides containing several lanthionine residues. They exhibit substantial antimicrobial activity against Gram-positive bacteria, including relevant pathogens. The production of the model lantibiotic nisin minimally requires the expression of the modification and export machinery. The last step during nisin maturation is the cleavage of the leader peptide. This liberates the active compound and is catalyzed by the cell wall-anchored protease NisP. Here, we report the production and purification of a soluble variant of NisP. This has enabled us to study its specificity and test its suitability for biotechnological applications. The ability of soluble NisP to cleave leaders from various substrates was tested with two sets of nisin variants. The first set was designed to investigate the influence of amino acid variations in the leader peptide or variations around the cleavage site. The second set was designed to study the influence of the lanthionine ring topology on the proteolytic efficiency. We show that the substrate promiscuity is higher than has previously been suggested. Our results demonstrate the importance of the arginine residue at the end of the leader peptide and the importance of lanthionine rings in the substrate for specific cleavage. Collectively, these data indicate that NisP is a suitable protease for the activation of diverse heterologously expressed lantibiotics, which is required to release active antimicrobial compounds.

## Introduction

Lanthipeptides are posttranslationally modified peptides that contain dehydrated amino acids and (methyl)lanthionine residues (Knerr and van der Donk, 2012; Arnison et al., 2013). Lantibiotics are those lanthipeptides that have significant antimicrobial activity, namely lanthipeptide classes I and II. Some lantibiotics show activity against clinically relevant bacteria in a concentration range comparable to antibiotics in use. Moreover, they can target multidrug resistant bacteria (Mota-Meira et al., 2000; Piper et al., 2009). The production of the model lantibiotic nisin (belonging to the class I lanthipeptides) by *Lactococcus lactis* requires the coordinated expression of 11 genes (Lubelski et al., 2008). Prenisin is produced as a linear prepeptide that undergoes dehydration and cyclization in a directional and alternating way (Lubelski et al., 2009) and is subsequently exported by a dedicated transporter (NisT). Outside the cell, the protease NisP cleaves off the leader peptide releasing mature nisin (Kuipers et al., 1993). In this process, the leader peptide of nisin serves as a recognition motif for the modification enzymes and the transporter and keeps the fully modified prenisin inactive until it is removed (Kuipers et al., 1993; van der Meer et al., 1993; Oman and van der Donk, 2010; Khusainov et al., 2011, 2013; Plat et al., 2011, 2013; Khusainov and Kuipers, 2012; Abts et al., 2013; Yang and van der Donk, 2013).

The proteases involved in the maturation of lanthipeptides recognize different cleavage sites. The type I lanthipeptide proteases, generally referred to as LanP, are subtilisin-like serine proteases. They can be secreted to the extracellular medium, like EpiP (Geissler et al., 1996), or remain in the cytoplasm, like PepP (Meyer et al., 1995), or be exported and bound to the cell wall, like NisP. In the maturation of subtilin, no specific protease has been found and the processing takes place outside the cell probably by diverse serine proteases (Corvey et al., 2003). The first lantibiotic protease with a resolved 3D structure, EpiP from *Staphylococcus aureus*, an analog of NisP, has been reported (Kuhn et al., 2014; Xu et al., 2014). On the other hand, in type II lanthipeptides, the protease domain is fused to the transporter and this protein cleaves behind a double glycine motif (Knerr and van der Donk, 2012). In type III lanthipeptides, the cleavage is not so specific and is mediated by a prolyl oligopeptidase (Völler et al., 2013).

The modification enzymes of the nisin biosynthesis gene cluster have been used to produce potent and stable variants of clinically relevant peptides, providing extensive information regarding the promiscuity of the modification machinery (NisBC) and the transporter (NisT) (Kuipers et al., 2004; Kluskens et al., 2005, 2009; Rink et al., 2005, 2007a,b; Moll et al., 2010; Bosma et al., 2011; van Heel et al., 2016). The production of modified prelantibiotics allows to obviate the requirement for immunity and can achieve higher yields, although it requires a later activation by cleavage of the leader peptide (Valsesia et al., 2007; Majchrzykiewicz et al., 2010; Montalbán-López et al., 2017). Moreover, NisRK expressed in diverse strains provides a widely used inducible protein expression system for Gram-positive bacteria (De Ruyter et al., 1996; Eichenbaum et al., 1998; Mierau and Kleerebezem, 2005).

The promiscuity of NisBTC and the development of an efficient production system for the modification and export of modified peptides enabled the production of putative lantibiotics from diverse bacteria in *L. lactis* (Majchrzykiewicz et al., 2010; van Heel et al., 2016). Moreover, this production system can be extended with additional enzyme modules (i.e., additional modification enzymes found in lantibiotic gene clusters) (van Heel et al., 2013) or with non-canonical amino acids (Zhou et al., 2016; Baumann et al., 2017; Zambaldo et al., 2017) that increase the repertoire of unusual amino acids that can be incorporated *in vivo* in peptides. These findings highlight the large potential of using Synthetic Biology principles in the production of novel lanthipeptides (for a review see Montalbán-López et al., 2012, 2017 and references therein). The versatility of the NisBTC system allows for the production of a large number of putative prelantibiotics. Thus, a protease capable of releasing the active lantibiotic during growth of the producer strain is indispensable for using high-throughput screening systems on novel peptides. Additionally, the production of modified prelantibiotics can achieve higher yields because no immunity is required being the only drawback the necessity of a suitable leader peptidase (Valsesia et al., 2007). Therefore, we tested the suitability of diverse commercial proteases for the cleavage of the leader peptide of nisin after maturation in various growth conditions. We compared their activity with that of the lantibiotic protease NisP, especially in view of the importance of the variable residues present behind the cleavage site, to establish the potential of NisP for various biotechnological applications.

**Figure 1:**
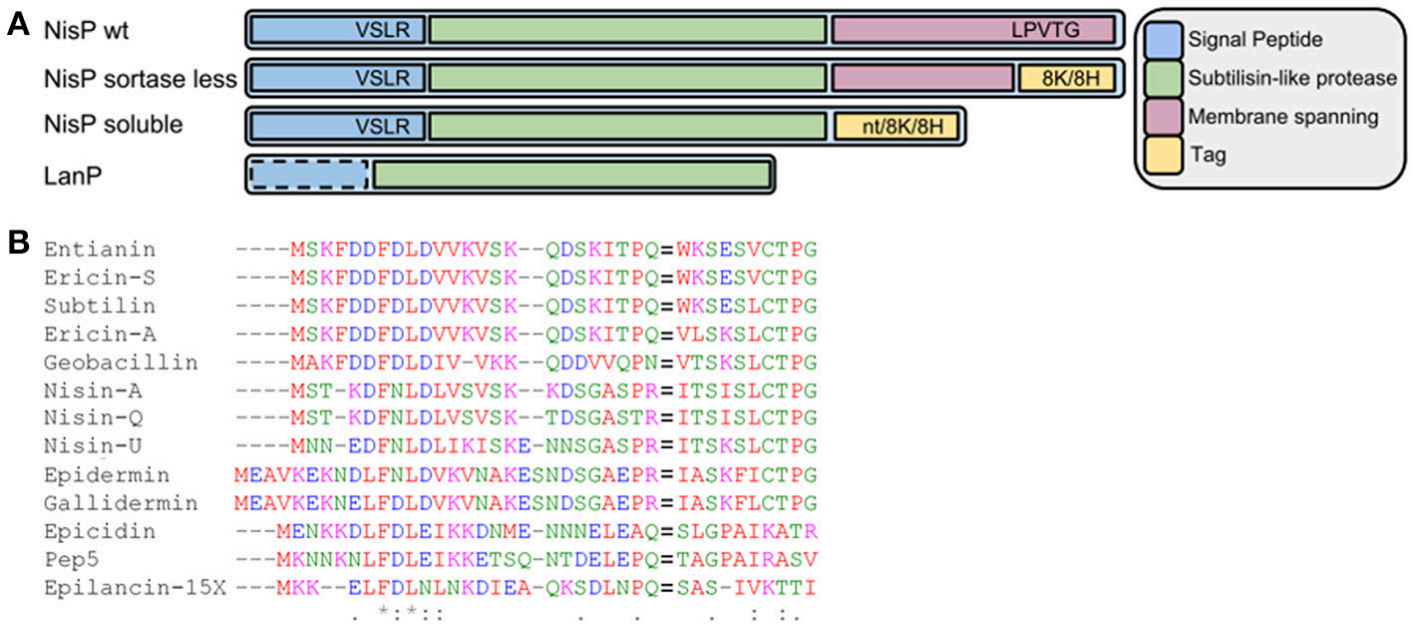
**(A)** Schematic view of wild-type NisP, NisP mutants generated in this work and other LanPs. 8H represents the 8-histidine tag, 8K represents the 8-lysine tag, and nt denotes no tag added. The dotted line around the signal peptide in LanP indicates that this part is not present in all LanP proteases. **(B)** Alignment of diverse type I lantibiotic peptides. The F(D/N)LD motif and the Pro-2 are highly conserved. The cleavage site is indicated with an equal to sign (=).

The nisin protease, NisP, is produced and exported to the outside of the cell, where it is anchored to the peptidoglycan via a sortase-catalyzed coupling and performs its function, although a fraction of the protease escapes anchoring (van der Meer et al., 1993; Siezen et al., 1995; Xu et al., 2014). It contains a typical N-terminal secretion signal, a protease domain, a self-cleavage C-terminal sequence and a sortase recognition sequence (Figure 1A). The maturation of NisP involves the release of the signal peptide and part of the prepeptide, likely by self-cleavage of the N-terminal sequence (van der Meer et al., 1993), as has been shown for an EpiP-analog (Kuhn et al., 2014). Although the role of NisP in the production of nisin is crucial, little is known about the specificity of the protease or the recognition sites present in prenisin that allow for the binding and cleavage of the leader peptide, although recently the influence of lanthionine rings on processing specificity has been reported (Lagedroste et al., 2017). Moreover, the fact that NisP is attached to the cell wall has prevented a detailed study of the specificity of NisP. Here, we present a systematic study of an engineered soluble variant of the lantibiotic protease NisP. This work will greatly facilitate the efficient production and activation of a wide variety of active lantibiotics with a cost-effectively produced protease.

## Materials and methods

### Bacterial strains and growth conditions

The bacterial strains and vectors used in this work are listed in Table 1. Lactococcal strains were grown in M17 (Oxoid), supplemented with 0.5% glucose (GM-17) at 30°C for genetic manipulation or in the same conditions, but in MEM for peptide production (Rink et al., 2005). *Escherichia coli* and *Micrococcus flavus* strains were grown in LB at 37°C, while shaking at 250 rpm. When appropriate, erythromycin and/or chloramphenicol (Sigma-Aldrich) were added at a final concentration of 5 μg/ml. Kanamycin (Sigma-Aldrich) was used at a final concentration of 20 μg/ml.

**Table 1:** Strains and vectors used in this work.

**Strain**	**Characteristics**	**References**
*Lactococcus lactis* NZ9000	*nisRK::pepN*	Kuipers et al., 1997
*Lactococcus lactis* NZ9700	Nisin producer	Kuipers et al., 1993
*Micrococcus flavus* B423	Sensitive strain	NIZO Food Research
*Escherichia coli* Rosetta Blue DE3	Expression host	Novagen
**Plasmid**	**Characteristics**	**References**
pET28b	Vector with the IPTG inducible P_T7_. Km^R^	Novagen
pNZ8048	Cm^R^ P_nisA_	De Ruyter et al., 1996
pNZE3-empty	Ery^R^	van Heel et al., 2013
pIL3BTC	Cm^R^ P_nisA_-*nisBTC*	Rink et al., 2005
pNZnisA-E3	Ery^R^ P_nisA_-nisA	Kuipers et al., 2004
pNZE3-Cys-less	C-terminal His-tagged Nisin (C7A C11A C18A C25A C27A) mutant. Ery^R^	Khusainov and Kuipers, 2013
pIL253	Ery^R^	Simon and Chopin, 1988
pNGnisTP	Cm^R^ P_nisA_-*nisTP*	Kuipers et al., 2004
pNZnisP-8H	P_nisA_-*nisP* with 8 histidines tag after the subtilisin-like domain. Cm^R^	This work
pNZnisP-8K	P_nisA_-*nisP* with 8 lysines tag after the subtilisin-like domain. Cm^R^	This work
pNZnisP-sol	P_nisA_-*nisP* with no tag fused after the subtilisin-like domain. Cm^R^	This work
pNZnisPsl-8H	P_nisA_-*nisP* sortase-less with 8 histidines tag. Cm^R^	This work
pNZnisPsl-8K	P_nisA_-*nisP* sortase-less with 8 lysines tag. Cm^R^	This work
pETNisP-sol	P_T7_-*nisP* with no tag fused after the subtilisin-like domain. Km^R^	This work
pETNisP-8H	P_T7_-*nisP* with 8 lysines tag after the subtilisin-like domain. Km^R^	This work
pETnisP-8K	P_T7_-*nisP* with 8 lysines tag fused after the subtilisin-like domain. Km^R^	This work
pNZE3nisA-C7A-ASPR	Nisin C7A mutant. Ery^R^	This work
pNZE3nisA-Cysless-ASPR	Nisin (C7A C11A C18A C25A C27A) mutant lacking all cysteines. Ery^R^	This work
pNZE3nisA-CAAAA-ASPR	Nisin (C11A C18A C25A C27A) mutant retaining only the first cysteine in the prepeptide. Ery^R^	This work
pNZE3nis-ringAless-ASPR	Nisin (T2V S3A S5A C7A) mutant, Ery^R^	This work
pNZE3nisA-VSLR	Nisin (A-4V P-2L) mutant containing a VSLR instead of the typical ASPR sequence in the leader, Ery^R^	This work
pNZE3nisA-C7A-VSLR	Nisin (A-4V P-2L C7A) mutant with a VSLR NisP cleavage site, Ery^R^	This work
pNZE3nisA-Cysless-VSLR	Nisin (A-4V P-2L C7A C11A C18A C25A C27A) mutant lacking all cysteines and with a VSLR NisP site, Ery^R^	This work
pNZE3nisA-CAAAA-VSLR	Nisin (A-4V P-2L C11A C18A C25A C27A) mutant retaining only the first cysteine in the prepeptide and with a VSLR NisP site, Ery^R^	This work
pNZE3nisA-ringAless-VSLR	Nisin (A-4V P-2L T2V S3A S5A C7A) mutant with a VSLR NisP site, Ery^R^	This work
pNZE3nisA-I1D	Nisin I1D mutant, Ery^R^	This work
pNZE3nisA-I1W	Nisin I1W mutant, Ery^R^	This work
pNZE3nisA-I1K	Nisin I1K mutant, Ery^R^	This work
pNZE3nisA-T2K	Nisin T2K mutant, Ery^R^	This work
pNZE3nisA-T2V	Nisin T2V mutant, Ery^R^	This work
pNZE3-DDDK	NisP cleavage site ASPR replaced by DDDK, Ery^R^	Plat et al., 2011
pNZE3-DDDDK	NisP cleavage site GASPR replaced by DDDDK, Ery^R^	Plat et al., 2011
pNZE3-AFNLD	Nisin D-19A mutant, Ery^R^	Plat et al., 2011
pNZE3-DANLD	Nisin F-18A mutant, Ery^R^	Plat et al., 2011
pNZE3-DFALD	Nisin N-17A mutant, Ery^R^	Plat et al., 2011
pNZE3-DFNAD	Nisin L-16A mutant, Ery^R^	Plat et al., 2011
pNZE3-DFNLA	Nisin D-15A mutant, Ery^R^	Plat et al., 2011
pNZE3-nis-V8	Nisin Z R-1E mutant, Ery^R^	This work
pNZE3-nis-Fx	Nisin Z (A-4I S-3E P-2G) mutant with IEGR replacing the ASPR NisP cleavage site, Ery^R^	This work
pNZE3-nis-Thr	Nisin Z (S-3V) mutant with AVPR replacing the ASPR NisP cleavage site, Ery^R^	This work
pNZE-nisΔ(23-34)	Nisin Δ(23-34) deletion mutant, Ery^R^	Rink et al., 2007b
pNZE3-1765	nisin leader peptide fused to the leaderless part encoded by spr1765, Ery^R^	Majchrzykiewicz et al., 2010
pNZE3-1766	nisin leader peptide fused to the leaderless part encoded by spr1766, Ery^R^	Majchrzykiewicz et al., 2010

*Cm^R^, chloramphenicol resistance; Ery^R^, erythromycin resistance*.

### Construction of expression vectors

Cloning steps were performed following standard protocols (Sambrook et al., 2001). The preparation of competent *L. lactis* cells and transformation were carried out according to Holo and Nes (1995). Restriction endonucleases and ligase were used as recommended by the provider (Thermo Fisher Scientific).

For the cloning of *nisP* variants, the gene was amplified from the genome of *L. lactis* NZ9700 using primers nisPbsphfwd and nisP8KXbarev or nisPbsphfwd and nisP8KSacIr for the addition of a 8-mer poly-lysine tag, nisPbsphfwd and nisP8HXbarev or nisPbsphfwd and nisP8HSacIr for the addition of a 8-mer poly-histidine tag, or nisPbsphfwd and solNisPcontrol for the production of an untagged soluble NisP (Supplementary Table 1). The amplification was performed using Phusion Polymerase (Thermo Fisher Scientific) following the provider's instructions. After amplification, the DNA was purified using the PCR cleaning kit (Roche) and digested with *Bsp*HI and *Xba*I or *Bsp*HI and *Sac*I and ligated in pNZ8048 digested with *Nco*I and either *Xba*I or *Sac*I. Similarly, the fragment was inserted into pET28b digested with *Nco*I and either *Spe*I or *Sac*I. The ligation mix was transformed into *L. lactis* NZ9000 or *E. coli* Rosetta Blue DE3. The nucleotide sequence of each gene was checked by sequencing with the primers listed in Supplementary Table 1.

For the construction of pNZE3nisA-CAAAA-ASPR, the nisin CAAAA coding gene (last 4 Cys replaced by Ala) was synthesized by GeneArt and cloned into pNZE3-empty (van Heel et al., 2013) as a *Bgl*II-*Hind*III fragment encoding the P_*nisA*_ promoter and *nisin-CAAAA*. For the construction of pNZE3nisA-Cysless-ASPR, Nisin Cys-less was amplified from pNZE3-Cys-less (Khusainov and Kuipers, 2013) using the primers pNZE3Emf and C-lessH6-less, digested with *Bgl*II and *Hind*III, and cloned into pNZE3-empty cut with the same enzymes. pNZE3nisA-C7A-VSLR was produced by round PCR of pNZnisA-E3 using the primers NisPC7A-rev and NisPC7A-fwd. pNZE3nisA-C7A-ASPR was produced by round PCR of pNZE3nisA-C7A-VSLR using the primers NisPC7A-rev and NisPC7A-ASPR-fwd The equivalent genes in which the end of the leader peptide was mutated from ASPR to VSLR were generated by round-PCR of each of the plasmids mentioned above with the primers nisVSLRfwd and nisVSLRrev.

The nisin variants with a mutation in the first two amino acids of the core peptide were produced by round PCR of pNZnisA-E3 using phosphorylated P-for as a forward primer in all cases and P-IK-Rev, P-KT-Rev, P-WT-Rev, P-DT-Rev or P-IV-Rev for Nisin A-T2K, Nisin A-I1K, Nisin A-I1W, Nisin A-I1D and Nisin A-T2V mutants, respectively.

### Gene expression and product purification

An overnight culture of *L. lactis* NZ9000 grown in GM17 with the desired plasmid(s) was diluted 50-times in preheated MEM and grown until an OD 600 nm of 0.4–0.6. At this moment, the culture was induced with 5 ng/ml of nisin (Sigma-Aldrich). Cells were harvested after 3 h of induction and the supernatants containing the protein of interest were further purified.

Trichloroacetic acid (TCA) precipitation was carried out according to Sambrook et al. (2001). The purification of nisin and its mutants in higher amounts was performed according to described protocols (Lubelski et al., 2009). When higher purity was required, the fractions collected were applied to a spherical C18 versaflash column (Supelco) previously equilibrated in 0.1% trifluoroacetic acid (TFA). The column was washed in three steps with 3 volumes of 33, 66, and 100% organic solvent (2:1 isopropanol:acetonitrile 0.1% TFA). After this step, the peptides were concentrated by freeze-drying.

For the purification of soluble truncated NisP, the producer cells were grown and induced at an OD 600 nm of 0.4–0.6 with either 5 ng/ml nisin or 1 mM IPTG depending on the producer strain being *L. lactis* NZ9000 or *E. coli* Rosetta Blue DE3, respectively. The cells were separated after 3 h induction by centrifugation at 6,000 rpm for 10 min at 4°C. The his-tagged variant NisP-8H was purified by affinity chromatography using a Ni-NTA fast flow resin (Qiagen). Briefly, the cell-free supernatant of *L. lactis* strains or the cell-lysate of *E. coli* strains was passed through a column previously equilibrated with binding buffer (20 mM phosphate buffer 0.5 M NaCl pH 8.0). The column was washed with 50 mM phosphate buffer 0.5 M NaCl 20 mM imidazole pH 8.0. NisP-8H was eluted from the column using 50 mM phosphate buffer 0.5 M NaCl 250 mM imidazole. NisP-8K was purified by cationic exchange chromatography using a Fast-flow SP-sepharose (GE Healthcare). The column was equilibrated with 5 column volumes of 20 mM phosphate buffer pH 6.5. The pH of the supernatant was adjusted to pH 6.5 and then passed through the column. After washing with 5 column volumes of 20 mM phosphate pH 6.5 0.5 M NaCl, the attached protein was eluted with 20 mM phosphate pH 6.5 1.5M NaCl.

The presence of NisP and its variants in elution fractions was assessed by checking the ability to activate prenisin in antimicrobial assays and/or by SDS-PAGE according to Laemmli (1970).

### Proteolysis of nisin and nisin mutants using NisP

Lyophilized nisin or its mutants were solubilized using a 0.05% acetic acid solution. Different buffers were prepared: 1 M HEPES, 1 M NaCl, 1 M HEPES 50 mM CaCl_2_, 1 M HEPES 50 mM MgCl2, 1 M MES, 1 M MES 50 mM CaCl_2_. In all cases, the pH was adjusted to 6.5 and mixed with each sample and then diluted 10 times. Additionally, 1 M Tris 50 mM CaCl_2_ pH 6.0 was tested. The pH was measured after mixing and corrected if necessary.

Alternatively, supernatants containing nisin mutant peptides produced after induction were divided into two fractions; one in which the pH was adjusted to 6.0 and the other one where the pH was the actual fermentation pH. They were incubated overnight at 37°C with or without His-tagged purified NisP at a ratio of 1000:1. After the incubation, the supernatants were precipitated using TCA and resuspended in 1/20 volume 0.05% acetic acid. For the mutants that were produced in lower amounts, larger volumes were induced and the peptides were purified by cationic exchange chromatography as described before and separated by reverse phase chromatography using a Jupiter 4 μm Proteo 90 Å 250 × 4.6 mm C12 analytical column (Phenomenex). The column was equilibrated in 20% organic solvent (acetonitrile 0.1% TFA) before the sample injection. It was washed for 5 min before applying a linear gradient from 20 to 50% organic solvent in 20 min. 1 μl of each collected peak was used for mass-spectrometry determination.

### MALDI-ToF mass spectrometry characterization

Mass spectrometry analysis of the samples was performed by matrix-assisted laser desorption/ionization (MALDI) time-of-flight (ToF) in an ABI Voyager DE Pro (Applied Biosystems) operating in linear mode as previously described using external calibration (van Heel et al., 2013). Briefly, 1 μl sample was spotted, dried and washed with 5 μL Milli-Q water, on the target. Next, 1 μl of α-cyano-4-hydroxycinnamic acid 5 mg/ml (Sigma-Aldrich) was spotted on the sample.

### Activity test

Activity tests were performed by well-diffusion assay as indicated by van Heel et al. (2013). *L. lactis* NZ9000 transformed with the appropriate plasmids or *Micrococcus flavus* were used as sensor strains as specified in each figure. The cleavage of nisin by NisP after SDS-PAGE was monitored washing the gel according to Bhunia et al. (1987) and covering with an overlay of GM17 soft agar inoculated with NZ9000 (pIL3BTC pNZnisA-E3) induced with 1 ng/ml nisin. The overlay was incubated for 16 h, after which the presence of inhibition zones was evaluated.

### Enzyme kinetic assays

In order to investigate the substrate specificity and kinetic parameters of NisP, a set of mutants of P4-P1 was created. We used wild-type prenisin (ASPR), nis-Peng (VSLR), nis-Thrombin (AVPR), and nis-Factor Xa (IEGR) as substrates. The concentration of NisP was determined by the BCA assay with bovine serum albumin as standard. The conditions of cleavage reaction were optimized with 100 mM Tris buffer containing 5 mM CaCl_2_ (pH 6.0). The reaction was stopped at the indicated times adding TFA to a final concentration of 1%. The reaction was performed in 100 μl with 6.5 ng/ml NisP at 37°C. 1% TFA was added to terminate the reaction at 5 different time points (5, 15, 30, 45, and 60 min). All the samples were analyzed by analytical RP-HPLC as indicated before (Mu et al., 2015) and measuring the absorbance at 205 nm. For each substrate concentration, the initial velocity was calculated on the basis of the peak area of released nisin vs. time. The kinetic parameters were determined by fitting the calculated enzyme activities at different substrate concentrations (ranging from 1 to 25 μM) to a linear regression curve on Lineweaver–Burk double reciprocal plot (Lineweaver and Burk, 1934).

## Results

### Cloning and expression of a soluble NisP variant

The fact that NisP is a cell wall anchored protease has hindered a thorough characterization and assessment of its biotechnological properties. In a first attempt to produce soluble NisP, we designed primers that hybridize partially to the sequence of NisP immediately upstream of the sortase recognition sequence in the C-terminus of NisP (LPVTG). We designed the primers nisP8KXbarev and NisP8HXbarev, which add a tail of 8-Lys or 8-His residues, respectively. These tags facilitate the purification of the protease and can also serve for the immobilization on different materials for high-throughput applications.

The supernatants of the strains NZ9000 (pNZnisPsl-8H) and NZ9000 (pNZnisPsl-8K) were tested for the presence of protease activity after 3 h of induction with nisin. In each case, 5 μl of the supernatant was mixed with 50 μl of prenisin obtained by TCA precipitation of induced NZ9000 (pIL3BTC pNZnisA-E3). Positive controls using trypsin or the supernatant of induced NZ9000 (pNGnisTP) and a negative control containing only prenisin were used. The presence of an appropriate protease in the mix will release active nisin, thus we can monitor the activity of NisP by measuring the antimicrobial activity against the sensitive strain *Micrococcus flavus*. We could observe antimicrobial activity only in the positive controls, thus indicating that the variant NisP protease, if produced, is not active (data not shown).

**Figure 2:**
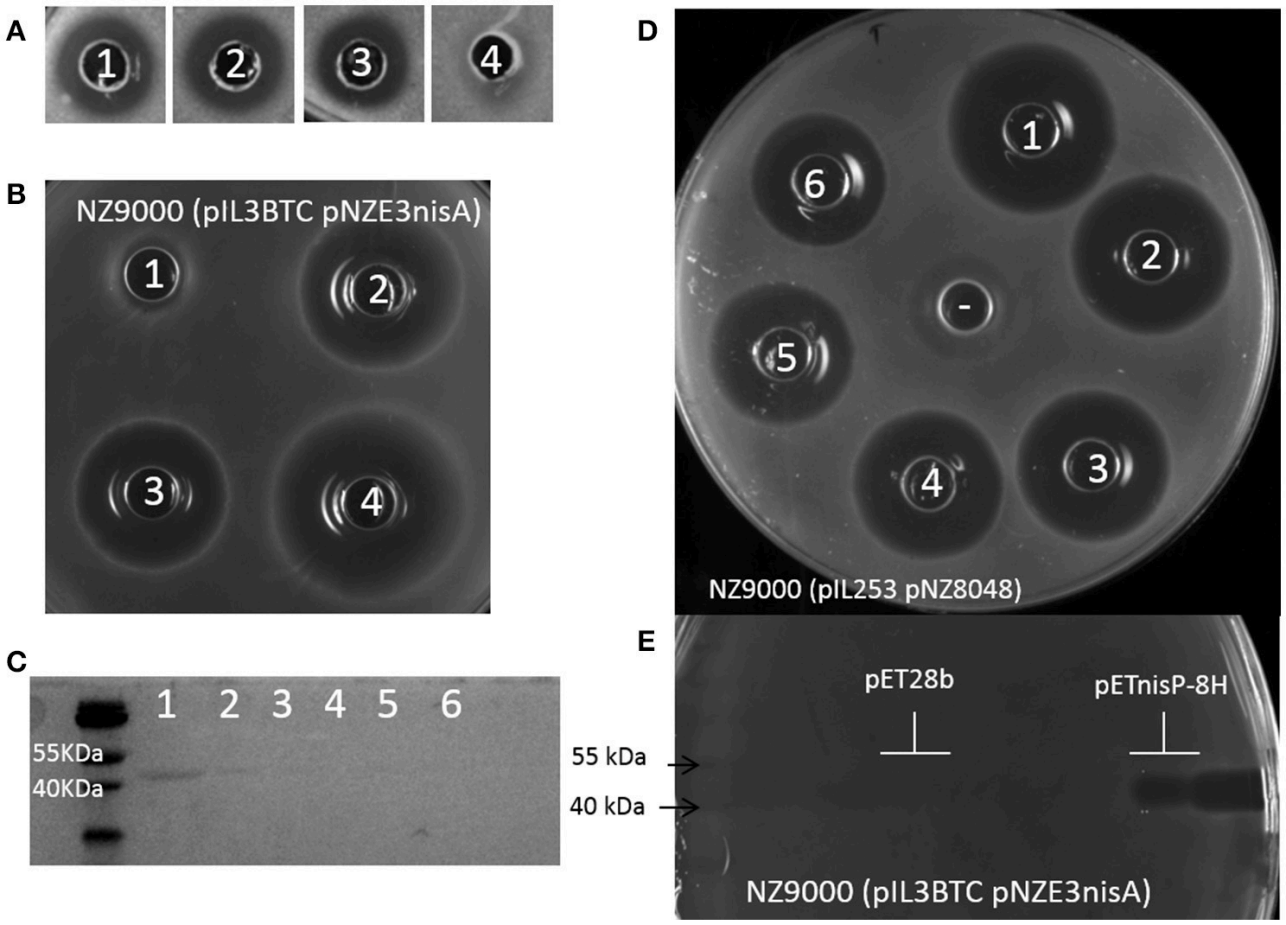
Activity of engineered NisP variants. **(A)** Activation of prenisin with supernatants of induced *L. lactis* NZ9000 transformed with pNZnisP-sol (1), pNZnisP-8H (2), pNZnisP-8K (3), or pNGnisTP (4), using *M. flavus* as an indicator strain. **(B)** Activity of NisP heterologously produced in *E. coli* Rosetta Blue DE3 using the prenisin producing strain *L. lactis* NZ9000 (pNZnisA-E3 pIL3BTC) both as producer and as sensitive strain. 20 μl of cell lysate of *E. coli* (pET28b) (1), *E. coli* (pETNisP-sol) (2) or *E. coli* (pETNisP-8H) (3) were added to the wells. 1 μl of NisP-8H purified from *L. lactis* NZ9000 (pNZnisP-8H) was used as a positive control (4). **(C)** SDS-PAGE of NisP-8H purified by affinity chromatography from *L. lactis* NZ9000 (pNZnisP-8H). Wells 1-6 contain 15 μl of each 2 ml-fraction collected. **(D)** Activation of prenisin by NisP-8H purified from *L. lactis* NZ9000 (pNZnisP-8H) using *L. lactis* NZ9000 (pNZ8048 pIL253) as a sensitive strain. Wells 1-6 correspond to a mix of 50 μl of supernatants of induced *L. lactis* NZ9000 (pNZnisA-E3 pIL3BTC) and 2 μl of purified NisP from the batch shown in **(C)**. **(E)** Activity test of NisP-8H from *E. coli* Rosetta Blue DE3 (pETnisP-8H) after separation by SDS-PAGE. *L. lactis* NZ9000 (pNZnisA-E3 pIL3BTC) was used both as producer and as sensitive strain.

In a second approach, we attempted to reduce the size of NisP, while keeping the active subtilisin-like domain intact. An alignment comparing different LanP proteases shows that NisP has a hydrophobic helix after the protease domain which is not present in other intracellular proteases (Velásquez et al., 2011). Thus, we decided to express a NisP variant that lacks the sortase recognition sequence and this motif. Similarly, we amplified the gene adding a C-terminal tag of either 8 histidines, or 8 lysines or no tag. We repeated the activity test with the supernatants of induced *L. lactis* NZ9000 (pNZnisP-sol), NZ9000 (pNZnisP-8H) and NZ9000 (pNZnisP-8K) (Figure 2A). In this case we were able to detect antimicrobial activity for all the soluble truncated NisP variants, with or without a tag attached to the C-terminus of the protein. Additionally, the size of the inhibition halo measured is larger in the samples activated with the soluble protease present in the supernatants of these three engineered strains compared to the samples activated with the supernatant of *L. lactis* NZ9000 (pNGnisTP), which produces as a major product wild-type cell-anchored NisP. This indicates that the amount of truncated NisP in the supernatants is higher than the amount of wild-type NisP exposed by the cells. These results demonstrate that the engineered NisP variants containing only the protease domain can be recovered from the supernatants. It also shows that C-terminally tagged NisP retains good activity.

The truncated NisP variants were also cloned in an *E. coli* expression vector, rendering pET28-NisP-8H, pET28-NisP-8K, and pET28-NisP-sol. The cell pellet obtained after induction of *E. coli* Rosetta Blue DE3 transformed with these vectors was disrupted using glass beads and used in an antimicrobial assay to activate prenisin. It was possible to activate prenisin using this fraction, showing the ability of *E. coli* to produce active truncated NisP (Figure 2B). Moreover, the supernatants of the induced cultures were also able to activate prenisin whereas the control did not.

### Purification of NisP and optimization of cleavage conditions

The strains *L. lactis* NZ9000 (pNZnisP-8H) and NZ9000 (pNZnisP-8K) were induced and the production of protease was monitored by SDS-PAGE of the diverse fractions collected during the purification by either affinity chromatography or cation exchange chromatography, respectively. We could clearly detect a highly pure band of approximately 42 kDa, which is the expected size after removal of the signal peptide during production (Figure 2C) with an average yield of 1 mg/L. The protease activity of each fraction was monitored by activation of prenisin (Figure 2D). Surprisingly, even in fractions for which the sensitivity of Coomassie blue staining was too low to detect the protease, activity could still be detected. Similarly, after induction of *E. coli* Rosetta Blue DE3 (pET28-NisP-8H) or (pET-NisP-8K) a protein of approximately 42 kDa was detected in the supernatants by SDS-PAGE. The gel was covered with a prenisin producing *L. lactis* strain demonstrating that this protein could activate prenisin and create an inhibition zone (Figure 2E).

The activity of NisP on wild-type prenisin was tested in different buffers based on Tris, HEPES or MES with or without calcium and magnesium and monitored by HPLC. We conducted the experiments in duplicate at 30 and 37°C. We found that the cleavage in 100 mM tris buffer pH 6.0 supplemented with 5 mM CaCl_2_ at 37°C was the optimal buffer for the reaction, although the protease was still reasonably active in the other conditions (data not shown).

### Prenisin specific cleavage using specific proteases in culture conditions

**Figure 3:**
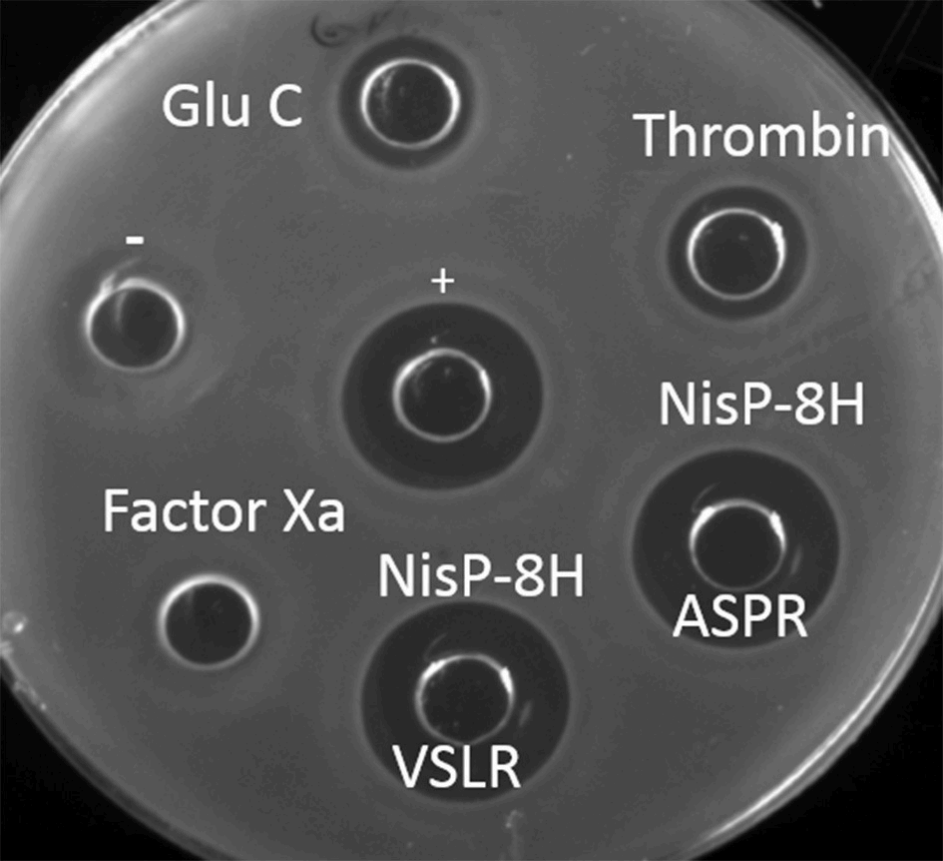
Activity of the proteases in crude supernatants. Antimicrobial assay against *L. lactis* NZ9000 (pIL253 pNZ8048) using different proteases. Each protease (1 μl) was mixed with supernatant containing 50 μl of its corresponding prenisin variant. Activity observed results from protease activity on the substrate releasing active nisin. + indicates a positive control of 50 μl nisin 10 ng/μl. − indicates a negative control of untreated prenisin. Supernatants of induced *L. lactis* NZ9000 (pIL3BTC pNZE3nis-V8) (Glu-C), NZ9000 (pIL3BTC pNZE3nis-Thr) (Thrombin), NZ9000 (pIL3BTC pNZE3nisA-ASPR) (NisP-8H ASPR), NZ9000 (pIL3BTC pNZE3nisA-VSLR) (NisP-8H VSLR), NZ9000 (pIL3BTC pNZE3nis-Fx) (Factor Xa) were mixed with 1 μl of the specific protease.

To further compare the versatility of NisP in culture conditions with various proteases frequently used in biotechnology, we mutated the last four amino acids in the leader peptide (P4-P1) of nisin (i.e., ASPR) to insert a factor Xa (IEGR) or a thrombin (AVPR) cleavage site. We also mutated the arginine in position P1 of the leader peptide into glutamic acid, creating a cleavage site for the endoprotease Glu-C. Additionally, the proposed self-cleavage sequence of NisP (VSLR) (Figure 1, van der Meer et al., 1993) was used to replace the ASPR sequence at the end of the nisin leader peptide. The supernatants of the prenisin producer strains containing these mutations were placed in a well on the agar plate and the specific proteases were added. The cleavage was evaluated by activity against NZ9000 (pIL253 pNZ8048), which is resistant to the erythromycin and chloramphenicol present in the supernatants of the substrate producer strains but sensitive to nisin. In these conditions, except for Factor Xa, all the proteases tested (thrombin, NisP and endoprotease Glu-C) were active (Figure 3).

### Specificity of NisP

**Figure 4:**
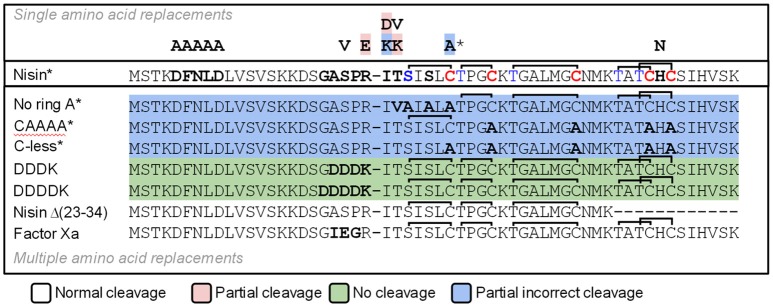
Schematic representation of the cleavage of nisin mutants using NisP-8H. Single mutants are indicated with one letter above wild-type nisin sequence. Mutants with several amino acid replacements are depicted in bold letters under wild-type nisin sequence. Lanthione rings are represented as a continuous line connecting Ser or Thr to Cys. The ^*^ indicates that this result was also obtained using the same variant with a VSLR cleavages site instead of the wild type ASPR site.

We designed a set of nisin mutants to investigate the specificity of NisP (Figure 4). For all these mutants, supernatants were collected after induction and divided into three fractions. NisP was added to two of these fractions, one maintaining the pH of the culture (4.5–5.0) and the other fraction was adjusted to pH 6.0. The third fraction was used as an untreated control. After cleavage, the masses of the peptides were monitored by MALDI-TOF (Table 2).

**Table 2:** Cleavage of nisin mutants using NisP on crude supernatants of the producer strain directly after fermentation or with the pH adjusted to 6.0.

***L. lactis* NZ9000 (pIL3BTC)**	**Expected**		**Measured**			**Dehydrations**
	**−NisP**	**+NisP**	**−NisP**	**+NisP**	**+NisP pH 6.0**	
pNZnisA-E3	5686.4	3354.2	5689.9 (+4.5)	3357.8 (+3.6)	3353.8 (−0.4)	8
		3372.2		3372.8 (+0.6)	3370.3 (−1.9)	7
		3390.2		3388.0 (−2.2)	3387.9 (−2.2)	6
pNZnisAC7A-ASPR	5654.3	3322.1	5663.9 (+9.6)	3322.7 (+0.6)		8
		3240.1		3340.0 (−0.1)		7
		3358.1		3357.3 (−0.8)		6
		3376.1		3374.9 (−1.2)		5
		3476.7		3478.8 (+2.1)		8 (+R)
		3494.7		3493.8 (−0.9)		7 (+R)
		3512.7		3512.2 (−0.5)		6 (+R)
		3208.9			3208.8 (−0.1)	8 (−I)
		3226.9			3225.5 (−1.4)	7 (−I)
		3244.9			3241.9 (−3.0)	6 (−I)
pNZE3nisA-ringAless-ASPR	5669.3	3337.1	5679.3 (+10.0)	3338.8 (+1.7)	3338.8 (+1.7)	4
		3355.1		3357.9 (+2.8)	3357.1 (+2.0)	3
		3142.8		3141.2 (−0.6)	3142.0 (−0.8)	3-IV
pNZE3nisA-CAAAA-ASPR	5540.1	3207.9		3207.8 (−0.1)	3210.3 (+2.4)	9
	5558.1	3225.9	5556.9 (−1.2)	3224.1 (−1.8)	3227.3 (+1.4)	8
		3243.9		3239.9 (−4.0)	3241.6 (−2.3)	7
pNZE3nisA-Cysless-ASPR	5526.1	3175.9	5528.0 (+1.9)	3175.9 (0)	3175.2 (−0.7)	9
		3193.9		3191.7 (−2.2)	3189.9 (−4.0)	8
		3332.1			3330.9 (−1.2)	9 (+R)
pNZE3nisA-VSLR	5730.4	3354.2	5728.6 (−1.8)	3353.0 (−1.2)	3353.7 (−0.5)	8
		3372.2		3368.9 (−3.3)	3366.8 (−5.4)	7
		3390.2		3385.9 (−4.3)	3390.9 (+0.7)	6
pNZE3nisA-C7A-VSLR		3322.1		3322.1 (0)	3322.9 (+0.8)	8
	5716.4	3340.1	5722.6 (+6.2)	3337.2 (−2.9)		7
		3478.3		3478.3 (0)	3479.1 (+0.8)	8 (+R)
		3496.3		3493.9 (−2.4)	3495.2 (−1.1)	7 (+R)
		3591.4		3590.2 (−1.2)		8 (+LR)
		3609.4		3608.6 (−0.8)		7 (+LR)
pNZE3nisA-ringAless-VSLR	5711.9	3337.1	5717.9 (+6.0)	3333.5 (−3.6)	3343.9 (+6.8)	4
		3223.9		3231.1 (+7.2)	3230.8 (+6.9)	4 (−I)
pNZE3nisA-CAAAA-VSLR	5602.2	3207.9	5596.2 (−6.0)	3207.3 (−0.6)	3207.8 (−0.1)	8
		3225.9		3223.4 (−2.5)	3223.9 (0)	7
		3243.9			3239.9 (−4.0)	6
pNZE3nisA-Cysless-VSLR	5552.1	3175.9		3177.8 (+1.9)	3166.7 (−9.2)	9
	5588.1		5583.6 (−4.5)			7
		3366.8		3361.1 (−5.7)	3365.5 (−1.3)	7 (+R)
		3445.2			3444.2 (−1.3)	8 (+VR)
		3649.4		3650.9 (+1.5)	3655.5 (+6.1)	7 (+VSLR)
pNZE3nisA-I1K	5737.4	3369.2	5741.6 (+4.2)	3369.9 (+0.7)	3369.6 (+0.4)	6
	5755.4	3387.2	5764.7 (+9.3)	3387.6 (+0.4)	3385.9 (−1.3)	5
		3405.2		3404.8 (−0.4)	3403.6 (−1.6)	4
		3525.4		3522.1 (−3.3)		8 (+R)
pNZE3nisA-T2K	5729.9	3373.2			3373.0 (−0.2)	7
	5747.9	3391.2	5746.2 (−1.7)	3396.4 (+5.2)	3391.0 (−0.2)	6
pNZE3-nisA-T2V	5702.4	3370.2		3370.1 (−0.1)	3770.0 (−0.2)	7
	5720.4	3388.2		3387.3 (−0.9)	3388.3 (+0.1)	6
	5738.4	3406.2		3404.5 (−1.7)	3405.6 (−0.6)	5
	5756.4		5756.4 (0)			4
	5774.4		5781.7 (+7.3)			3
pNZE3-nisA-I1D	5706.3	3374.1		3373.4 (−0.7)	3373.7 (−0.4)	7
	5724.3	3392.1		3391.2 (−0.9)	3391.7 (−0.4)	6
	5742.3	3410.1	5746.6 (+4.3)		3407.7 (−2.4)	5
pNZE3-nisA-I1W	5803.4	3427.1	5808.5 (+4.1)	3425.8 (−1.3)	3424.7 (+0.6)	8
	5821.4	3445.1	5820.9 (−0.5)	3444.1 (−1.4)	3443.9 (−1.2)	7
	5839.4	3463.1		3460.9 (−2.2)	3464.2 (+1.1)	6
	5857.4		5855.3 (−2.1)			5
pNZE3-nisA-V8	5636.3	3331.1	5646.4 (+10.1)	3331.3 (+0.2)	3334.3 (+3.2)	8
		3349.1		3348.0 (−1.1)	3353.4 (+4.3)	7
pNZE3-nisA-Thr	5675.4	3331.1	5685.4 (+10.0)	3330.5 (−0.6)	3326.9 (−4.2)	8
		3349.1		3350.0 (+0.9)	3350.1 (+1.0)	7
pNZE3-nisA-FX	5707.4	3331.1	5709.5 (+2.1)	3334.7 (+3.6)	3332.9 (+1.8)	8
	5725.4		5727.6 (+2.2)			7
pNZE3nisA-DDDK	5748.3	3354.2	5744.9 (−3.4)		5743.2 (−5.1)	8
	5766.3			5763.2 (−3.1)		7
pNZE3-DDDDK	5806.3	3354.2	5804.1 (−2.2)	5812.3 (+6.0)		8
	5824.3				5824.6 (+0.3)	7
pNZE3-AFNLD	5642.4	3354.2	5643.4 (+1.0)	3355.1 (+0.9)		8
		3372.2		3378.8 (+6.6)	3371.8 (−0.4)	7
pNZE3-DANLD		3354.2		3353.0 (−1.2)	3350.7 (−3.5)	8
	5628.3	3372.2	5629.9 (+1.6)	3370.1 (−2.1)	3367.8 (−4.4)	7
		3390.2		3386.1 (−4.1)	3384.3 (−5.9)	6
		3408.2		3402.2 (−6.0)	3400.5 (−7.7)	5
pNZE3-DFALD	5643.4	3354.2	5647.8 (+4.4)	3351.1 (−3.1)	3350.9 (−3.3)	8
		3372.2		3368.0 (−4.2)	3371.0 (−1.2)	7
		3390.2		3390.8 (+0.6)	3394.2 (+4.0)	6
pNZE3-DFNAD	5644.3	3354.2	5652.9 (+8.6)		3357.3 (+3.1)	8
		3372.2		3370.1 (−2.1)	3374.3 (+2.1)	7
		3390.2		3387.3 (−2.9)		6
pNZE3-DFNLA	5642.4	3354.2		3357.7 (+3.5)	3357.5 (+3.3)	8
	5660.4	3372.2	5656.0 (−4.4)	3371.9 (−0.3)	3371.2 (+1.0)	7
		3390.2		3386.8 (−3.4)		6
pNZE-nis Δ(23-34)		2139.0		2138.5 (−0.5)	n.d.	5
	4489.9	2157.0	4485.8 (−4.1)	2154.6 (−2.4)		4
		2175.0		2170.7 (−4.3)		3

*−, not addition of NisP; + addition of NisP; n.d., not determined*.

*The mass difference between the expected and the measured masses is displayed in brackets for each one of the possible dehydration statuses considered. In the case of misscleavage, the additional or lacking amino acids are displayed in brackets in the dehydration column together with the number of dehydroamino acids*.

### Lanthionine ring-impaired nisin mutants

We designed variants of nisin containing only the first ring (nisin-CAAAA), lacking the first ring (nisin-C7A) and lacking all the rings (nisin-Cys-less). In order to discard an abnormal ring formation between Cys11 and an N-terminal dehydrated residue other than Thr8 in the mutant nisin-C7A, we designed the mutant nisin-ringA-less, where the first dehydratable residue is Thr8 and therefore we abolish the possibility of any aberrant ring formation. Additionally, we mutated the residues P4 to P1 in the leader peptide of nisin to create a VSLR cleavage sequence in nisin, nisin-CAAAA, nisin-C7A, nisin-ringA-less and nisin-Cys-less. We observed that wild-type nisin was fully cleaved, independently of the cleavage sequence at the end of the leader peptide being ASPR or VSLR. In the other cases, although most of the cleavage took place in the right position, we were able to detect masses corresponding to aberrant cleavage by NisP (Table 2).

The mutant lacking all the cysteines (nisin-Cys-less) was produced in a very low amount, irrespectively of the cleavage site present. After the cleavage of nisin-Cys-less, diverse peaks with a mass corresponding to the cleaved and dehydrated peptide were detected with a small fraction of incorrectly processed nisin containing additional residues from the leader peptide (Table 2). Similarly, in the mutants nisin-CAAAA, nisin-C7A and nisin-ringA-less, small peaks corresponding to peptides cleaved at abnormal P1 or P2 positions in the leader peptide were also observed (Table 2). In the mutants nisin-C7A and nisin-ringA-less, a small fraction of nisin lacking the first amino acid(s) was also identified, suggesting cleavage at alternative positions.

### Cleavage site mutants

Various amino acid residues were replaced at the N-terminus of the nisin core peptide (positions P1′ and P2′) to investigate the influence of these residues on the cleavage by NisP. We engineered positive and negative charges (nisin I1K, nisin T2K, and nisin I1D), a bulky amino acid (nisin I1W) and a hydrophobic amino acid in the second position of nisin core peptide replacing the dehydrobutyrine residue (nisin T2V) (Table 2). In all P1′ and P2′ mutants, we could detect inhibition of growth of sensor bacteria after activation with NisP. This indicates that these positions can tolerate a large variety of amino acids of very different nature and can still be cut by the specific protease and retain antimicrobial activity. It was possible to detect a small fraction of uncleaved peptide after the incubation of the mutant nisin I1D with the protease, which indicates reduced cleavage efficiency. The mutant nisin I1K rendered a small fraction of mature nisin with a mass difference consistent with the presence of Arg P1 in the mature peptide, indicating that the peptide is incorrectly cleaved behind ASP.

Additionally, we tested the efficiency of NisP to cleave other protease recognition sequences, namely Factor Xa, thrombin, endoprotease Glu-C, and enterokinase (DDDK and DDDDK sequences) (Table 2). NisP was able to cleave when a factor Xa or thrombin site was present. Surprisingly, the nisin mutant with a glutamic acid in the P1 position of the leader peptide (nisin V8) was cleaved, although only partially since uncut peptide was still visible after the experiment. The final amount of undigested peptide in case of the mutant nisin V8 was higher when the cleavage was performed at pH 6. The enterokinase sequences DDDK or DDDDK were not cleaved by NisP in the conditions tested. Noticeably, no unspecific cleavage in the core peptide of any of the tested mutants was observed in the conditions tested.

### Nisin mutants in the (D)FNLD box

Previously reported single alanine replacements of the characteristic DFNLD motif of type I lantibiotics leader peptide were studied (Plat et al., 2011). In all cases, cleavage was taking place normally as shown by mass-spectrometry (Table 2) and antimicrobial assays (data not shown) were consistent with previous reports (Plat et al., 2011).

### Cleavage of different lantibiotic substrates

**Figure 5:**
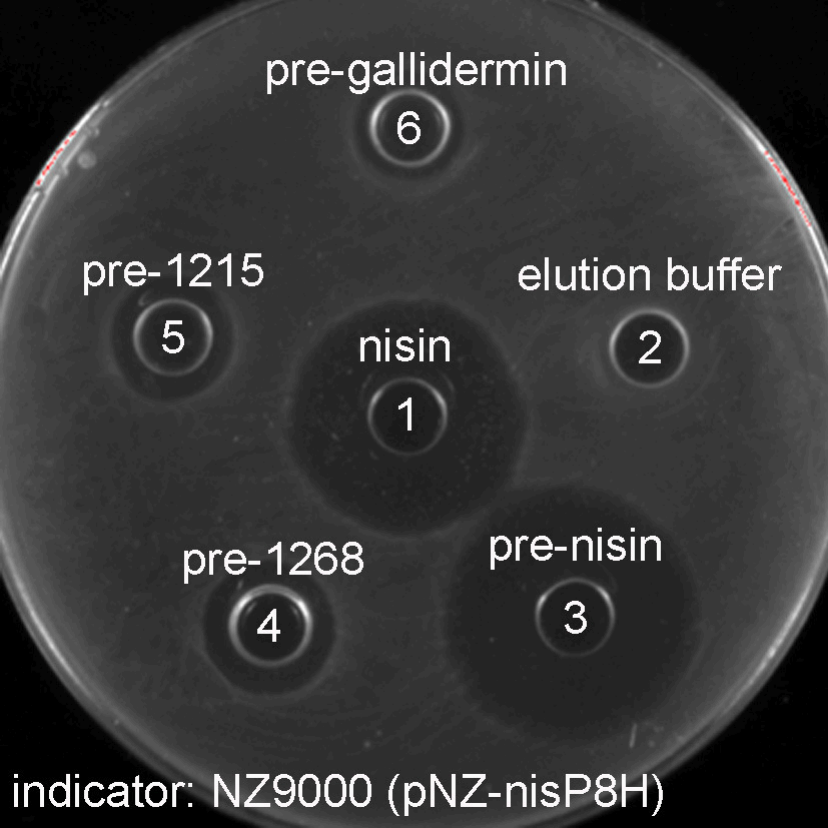
Antimicrobial activity of purified modified lantibiotics gallidermin, 1215 and 1268 against *L. lactis* NZ9000 (pNZnisP-8H). (1) Commercial nisin; (2) elution buffer used during the purification, (3) purified prenisin; (4) pre-1268; (5) pre-1215; (6) pregallidermin.

The substrate tolerance of soluble NisP was tested using a set of different lantibiotics. These were produced using the nisin modification machinery encoded in NZ9000 (pIL3BTC pNZE3-mutant) and purified by cationic exchange chromatography. For this purpose, NZ9000 (pNZ-nisP-8H) was used as a sensor strain and tested using gallidermin (van Heel et al., 2013) and two lantibiotics detected in the genome of *Streptococcus pneumoniae*, PneA1 and PneA2 encoded in the genes *spr1765* and *spr1766*, respectively, which were heterologously expressed in *L. lactis* fused to the nisin leader peptide (Majchrzykiewicz et al., 2010) (Figure 5). We could clearly determine that in the absence of NisP, none of the prelantibiotics tested, not even prenisin, was active against the sensor strain (data not shown). The activity was restored when a strain producing soluble NisP was employed. We could also notice that the NisP producing strain showed increased sensitivity to nisin (data not shown).

### Kinetic characterization of NisP

**Figure 6:**
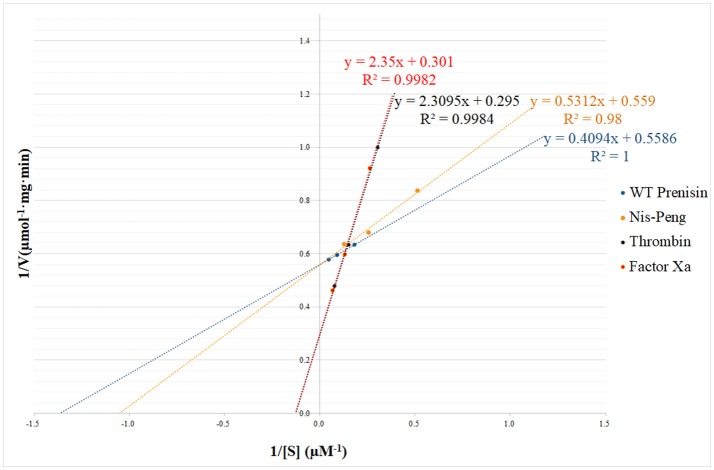
NisP kinetic parameters determination using Lineweaver–Burk plot. The substrate concentrations ranged between 1 and 25 μM.

The substrate specificity and kinetic parameters of engineered soluble NisP were investigated by using WT prenisin (ASPR), nis-Peng (VSLR), nis-Thrombin (AVPR), and nis-Factor Xa (IEGR) as substrates. The *K*_*m*_ and *V*_*max*_ values were determined using Lineweaver–Burk plots (Figure 6). When wild-type prenisin was used as substrate, NisP exhibited the highest catalytic efficiency (*K*_*cat*_*/K*_*m*_ = 1.71 × 10^6^ M^−1^s^−1^) and affinity (*K*_*m*_ = 0.73 μM). In comparison with wild-type prenisin (ASPR), Nis-Peng (VSLR) showed a slightly increased *K*_*m*_ value, an identical *K*_*cat*_ value and a comparable *K*_*cat*_*/K*_*m*_ values (Table 3). For nis-Thrombin (AVPR) and nis-Factor Xa (IEGR), the 5–6-fold decrease in *K*_*cat*_*/K*_*m*_ was ascribed to a 10–11-fold increase in *K*_*m*_ and 2-fold increase in *K*_*cat*_ compared with that of wild-type prenisin (Table 3). In summary, soluble NisP displayed the highest catalytic efficiency to wild-type prenisin (ASPR), followed by Nis-peng (VSLR). A 5–6-fold decreased catalytic efficiency was observed when Thrombin (AVPR) and Factor Xa (IEGR) cleavage sites were engineered.

**Table 3:** Kinetic characterization of the cleavage of several nisin leader peptide mutants.

**Prenisin variants**	**K_m_ (μM)**	**Vmax (μmol/mg/min)**	**K_cat_ (s^−1^)**	**K_cat_/K_m_ (M^−1^s^−1^)**
WT Prenisin (ASPR)	0.73 ± 0.08	1.79 ± 0.10	1.25 ± 0.07	1.71 × 10^6^
Nis-Peng (VSLR)	0.95 ± 0.08	1.79 ± 0.17	1.25 ± 0.11	1.32 × 10^6^
Thombin (AVPR)	7.83 ± 0.27	3.39 ± 0.20	2.37 ± 0.14	3.03 × 10^5^
Factor Xa (IEGR)	7.81 ± 0.01	3.32 ± 0.10	2.33 ± 0.07	2.98 × 10^5^

*Data indicate the mean value ± standard deviation*.

## Discussion

### Active soluble NisP can be produced and purified

During the production of nisin, the protease NisP is exported and anchored to the cell wall in a sortase-mediated manner. In previous attempts to characterize the specificity of NisP, the *in vivo* cleavage was based on the detection of the antimicrobial activity and the detection of mature lantibiotic compared to that of the prelantibiotic (van der Meer et al., 1994). The activity *in vitro* could be confirmed using membrane preparations of cells expressing NisP bound to the cell wall, which limited the assays. Therefore, we engineered a soluble NisP variant that could facilitate this task. Our initial hypothesis was that the removal of the sortase recognition sequence in NisP would abolish the binding, thus allowing for the purification of unbound protease in the supernatants (Figure 1). This approach failed to produce soluble NisP, maybe because NisP cannot fold properly or its solubility is reduced. Therefore, a second set of NisP mutants, trimmed at the C-terminus to remove the cell wall spanning helix, were designed. In this case, it was possible to produce detectable amounts of active NisP with the expected size in the supernatants. The activation of prenisin is unambiguously due to the action of this protease, since the strain with the empty vector was not able to activate prenisin. We also show that the purified protein could activate prenisin following SDS-PAGE electrophoresis, producing an inhibition halo around the band of the purified protease. These active NisP variants leave the subtilisin-like serine protease domain intact, and are more similar in size to other LanP-like proteases, which are naturally produced as soluble forms (EpiP, Geissler et al., 1996 or PepP, Meyer et al., 1995) or have been heterologously produced as soluble proteases (ElxP, Velásquez et al., 2011). Notably, *E. coli* was also able to produce mature NisP with the expected size in the supernatants (data not shown). This indicates that the secretion signal is correctly processed in *E. coli* and that the self-activation of NisP can happen. In previous reported attempts to express the full length NisP in *E. coli*, no detection of the protease by Coomassie staining was possible. However, isotope-labeled methionine showed the production of a processed NisP with a size consistent with the removal of the signal sequence (van der Meer et al., 1993), as was observed in our study. This cell wall attached NisP could activate prenisin when cell extracts of induced *E. coli* were used (van der Meer et al., 1993). Importantly, the activity of NisP was not abolished by the addition of a C-terminal poly-histidine tag or a poly-lysine tag.

### NisP tolerates mutations at the N-terminus of mature nisin

A homology modeling study on NisP based on the comparison with other proteases from the same family predicted an active site with a binding pocket that could fit 6 amino acids (P4, P3, P2, P1, P1′, and P2′) (Siezen et al., 1995). It predicts a strong interaction between the arginine in the position P1 with the protease mediated by electrostatic interactions with two aspartates in the enzyme that limits the amino acids in the positions P1′ and P2′ to be small hydrophobic amino acids (Siezen et al., 1995).

The presence of amino acids of diverse nature in the positions P1′ and P2′ of nisin shows that these residues can be mutated without altering the cleavage by NisP. It was anticipated that the binding of dehydrobutyrine in position P2′ was less important than the interaction of Ile in position P1′ with a hydrophobic groove in NisP (Siezen et al., 1995). Some mutants like I1W were previously shown to be cut by NisP using only the antimicrobial activity as evidence (Kuipers et al., 1995). Here we show that it is fully processed under the experimental conditions used. We could observe that an additional positive charge in the mutant I1K produced a small additional peak detected by mass-spectrometry consistent with a cleavage between positions P2 and P1, suggesting that the accumulation of positive charges favors a non-specific cleavage by NisP. Bulky or charged amino acids are well tolerated and the dehydrobutyrine in the second position, or a previously engineered dehydroalanine (Kuipers et al., 1995), are not the requirement for cleavage (Siezen et al., 1995).

### Lanthionine rings in nisin are not essential for cleavage by NisP

Additional studies have suggested the requirement of lanthionine rings as a prerequisite for the substrate recognition (Kuipers et al., 2004). We engineered various mutants with altered lanthionine rings. NisP is supposed to cut in the VSLR+QP sequence in its N-terminus (van der Meer et al., 1993) and VSVR+S at the C-terminus (Xu et al., 2014), which lack lanthionine rings. Therefore, we hypothesize that a higher tolerance could be expected for mutants containing the engineered VSLR site in the leader peptide. Thus, we engineered in all the mutants with modified lanthionine ring topology the self-activation site proposed for NisP, the VSLR cleavage sequence. In previous work, a nisin mutant (A-4V,P-2L,I1Q,T2P, positions P4, P2, P1′ and P2′ according to Schechter-Berger nomenclature), containing the whole self-cleavage site of NisP was previously reported (van der Meer et al., 1993; Plat et al., 2011). We show here that VSLR+IT is also cleaved by NisP. The cleavage pattern observed in the mutants with different number of rings shows that the presence of lanthionine is not essential for the cleavage, since nisin Cys-less seems to be cleaved and so is nisin CAAAA. A similar situation was observed with the cleavage *in vitro* of unmodified pre-epilancin 15X and unmodified pre-epidermin with the specific proteases ElxP and EpiP, respectively (Geissler et al., 1996; Velásquez et al., 2011). This is in disagreement with previous work using whole cells expressing cell-attached NisP that suggests the requirement of lanthionine rings for the cleavage reaction (Kuipers et al., 2004). This discordance can be due to the amount of NisP expressed at the cell surface which could be too small compared to the addition of purified NisP, the additional replacements in nisin (S-6P, P-2L, positions P6 and P2, respectively) and/or the shorter incubation time used. It has been already shown that the binding affinity can be much higher for the fully modified prelantibiotic than for the unmodified one (Geissler et al., 1996; Lagedroste et al., 2017). Our results also indicate that although the lanthionine rings are not essential for the cleavage, they might favor the correct interaction with the protease and positioning of the substrate in the active site since small amounts of prenisin with either additional residues of the leader peptide or lacking some amino acids of the core peptide were visible, indicating occasional misprocessing. NisP production involves self-activation after the N-terminal signal peptide and cleavage at the C-terminus for optimal nisin production. Thus the promiscuity demonstrated in this study is also related to NisP expression itself (which recognizes different (auto-)processing sites). This can be exploited for specific peptide cleavage at various slightly different sites, when producing variant lantibiotics behind the nisin leader peptide (Supplementary Figure 1).

### NisP cleaves in all tested protease sites, except in the two enterokinase sites

As mentioned before, the active site of NisP interacts with the last 4 residues of the leader peptide (Siezen et al., 1995). We modified these last positions in the leader peptide of nisin generating a set of diverse protease substrates. In such a way, we could investigate the specificity of NisP and simultaneously compare NisP with other proteases for the cleavage of the leader peptide in molecules modified using nisin biosynthesis machinery (*vide infra*).

The diverse protease sites engineered in the leader peptide prove that, except for the two enterokinase sites, all the other cleavage sequences tested could be cleaved by NisP, albeit with different efficiencies. Our K_cat_/K_m_ obtained for wild-type nisin is in line with previous results in literature, even though a different buffer is used (Lagedroste et al., 2017). The largest differences are noticed in the kinetic parameters of nis-Thrombin and nis-Factor Xa, with a 5–6-fold decrease in the catalytic efficiency (Table 3). Although the cleavage efficiency in these cases is reduced, this can be counteracted using prolonged incubation times or a higher enzyme dose, to yield full cleavage (data not shown). It is not surprising that the very similar thrombin site can be cleaved (AVPR vs. ASPR in the wild-type) although the reduction in the catalytic efficiency points at the favored cleavage with a hydrophilic residue in the P3 position that can interact with the solvent out of the protease active site (Siezen et al., 1995). The replacement of ASPR with the factor Xa cleavage site IEGR introduces additional changes and a negatively charged residue, but is still rather efficiently cleaved by NisP. The presence of negative residues in position P3 is common in other type I lantibiotics processed by a LanP enzyme such as gallidermin or epidermin. In contrast, the mutant A-4D (van der Meer et al., 1994) is not fully cleaved. The predicted interaction partner in the enzyme is a small hydrophobic pocket and therefore the mutation A-4D could not fit well into this cleft (Siezen et al., 1995). Additionally, a weak hydrogen bond interaction between Ser P3 and NisP was modeled, whereas in EpiP the presence of lysine residues around the catalytic site could favor a stronger interaction with the negative charge in epidermin leader peptide (Siezen et al., 1995). Our results support the idea that a small hydrophobic amino acid in the P4 position and a polar or charged amino acid in the P3 position may be important for the correct positioning of the leader peptide into the active site of NisP.

The highly conserved proline in the P2 position in the leader peptide of type I lantibiotics could play an important role in determining the local structure of the leader peptide and facilitating the access of the protease active site to cleave the mature prelantibiotic. It is also related to production levels and transport (Plat et al., 2017). Our data and the self-cleavage sequences in NisP and several other Pro mutants in the position P2 (van der Meer et al., 1994; Kuipers et al., 2004; Plat et al., 2011; Xu et al., 2014) clearly shows that proline is not essential for the cleavage (Figure 1B).

The highly conserved arginine in the P1 position establishes a strong ionic interaction with aspartate residues in the model structure of NisP (Siezen et al., 1995). This position shows certain flexibility, although the replacements R-1Q, which is present in other type I lantibiotics such as subtilin, Pep5 or epilancin 15X, and the mutation R-1E present in the mutant nisin V8, dramatically reduces the cleavage efficiency (van der Meer et al., 1994 and this work).

### Single alanine mutations in the (D)FNLD box do not hamper cleavage by NisP

The (D)FNLD box in lantibiotic leader peptides has been shown to be essential for the optimal interaction with the modification enzymes in nisin biosynthesis machinery and other type I lantibiotics (Lubelski et al., 2009; Khusainov et al., 2011, 2013; Plat et al., 2011, 2013; Knerr and van der Donk, 2012; Abts et al., 2013). Previous works reported the impact of various mutations in this region on the modification extent of nisin monitored by mass-spectrometry and activity tests. However, the activity was measured after activation with trypsin (Khusainov et al., 2011, 2013; Plat et al., 2011). We considered this DFNLD motif as a possible recognition sequence also for the protease NisP. This was previously considered in the homology modeling study in view on unaligned fragments of NisP that were considered important for the binding to the substrate prenisin (Siezen et al., 1995). All the single alanine replacements we tested as substrate for NisP could still be cleaved correctly. This suggests that either this is not a recognition site for NisP or that single alanine mutations are not enough to disrupt the interaction. Similarly, replacements in the region between the FNLD box and the ASPR cleavage sequence of the leader had no great impact on the production of nisin, as was published before (van der Meer et al., 1994).

### NisP as a tool to release diverse lanthionine-containing peptides

The plug-and-play production system for lantibiotics developed in the last years constitutes a robust platform for the production of diverse lanthionine-containing peptides (Rink et al., 2005; Majchrzykiewicz et al., 2010; van Heel et al., 2013, 2016). Moreover, prelantibiotics have no biological activity and higher yields can be achieved (Valsesia et al., 2007). Thus, a leader peptidase with broad specificity for the peptide moiety behind the cleavage site is desired. A cheap peptidase capable of working directly in culture broth is preferred since this can facilitate efforts during high-throughput screening approaches. NisP fulfills these two criteria and constitutes a valuable tool for the cleavage of the leader peptide in the production of lanthionine-stabilized peptide hormones (Kluskens et al., 2009) as well as fully modified non-cognate prelantibiotics (Figure 5). The increased sensitivity compared to the plasmid free strain can be due to stress caused by the production of the protease (data not shown). The attempts to express the lantibiotic protease ElxP in the wild-type producer also caused toxicity issues (Ortega et al., 2014).

Various proteases are commonly used for the cleavage of recombinant proteins with an affinity tag attached, such as TEV protease, enterokinase, Factor Xa, or thrombin. Some of them cleave at the end of their recognition sequence, whereas some others cleave in between, therefore adding extra residues after the cleavage site (i.e., TEV protease). The latter proteases are disadvantageous for our purposes since they leave some amino acids behind that cannot be removed. Factor Xa can tolerate most amino acids behind the cleavage sequence I(D/E)GR with the exception of lysine and proline. However it needs very controlled conditions and commonly cleaves incompletely making its biotechnological application costly. Thrombin, although it cleaves in the middle of the recognition sequence, shows some flexibility in the residues behind the cleavage site and tolerates different hydrophobic amino acids.

We tested different proteases and showed that NisP, the endoprotease Glu-C, thrombin and trypsin are able to cleave in culture conditions. Factor Xa could not activate prenisin in these conditions. Trypsin and the endoprotease Glu-C are very unspecific, since they can cleave behind positive or negative residues within the propeptide, respectively. This is a limitation to their applicability in many cases. However, the endoprotease Glu-C could be suitable in specific cases due to the low abundance of negative residues in lantibiotics and the pH conditions during cleavage can favor the cleavage selectivity for either aspartate or glutamic acid.

## Conclusions

Taken together, the results presented here suggest that the protease NisP has greater substrate tolerance than previously anticipated. The exact recognition motif in the leader peptide of nisin for the binding of NisP is not yet completely resolved, although our results agree with the predicted ionic interaction between Arg in position P1 and NisP as a requirement for efficient cleavage and confirm that the presence of lanthionine rings is not mandatory for the cleavage. The determination of the structure of NisP and the kinetics of the cleavage reaction of the different mutants could surely shed light on the residues more directly involved in the binding to NisP. These insights should help to expand the biotechnological potential of NisP as a general tool for the cleavage of proteins with and without lanthionine residues. Our results also show that among all the proteases tested, NisP is the most suitable and inexpensive candidate for the activation of diverse lantibiotics or thioether-stabilized peptides produced with the nisin leader peptide and the modification machinery of nisin.

## Author contributions

Planned and conceived the experiments: MM-L, AvH, and OK. Performed the experiments: MM-L and JD. Analyzed the experiments: MM-L, JD, AvH, and OK. Drafted the manuscript and contributed to the data interpretation: MM-L, JD, AvH, and OK. All authors read, critically revised and approved the final manuscript.

### Conflict of interest statement

The authors declare that the research was conducted in the absence of any commercial or financial relationships that could be construed as a potential conflict of interest.

## Supplementary material

Supplementary Figure 1 Schematic view of a possible workflow for the biotechnological application of NisP using nisin as an example.
