# Supplementary figures and images for: Specificity and Application of the Lantibiotic Protease NisP
Source: Front Microbiol. 2018 Feb 9;9:160. doi: 10.3389/fmicb.2018.00160 (PMC5812297; doi:10.3389/fmicb.2018.00160)

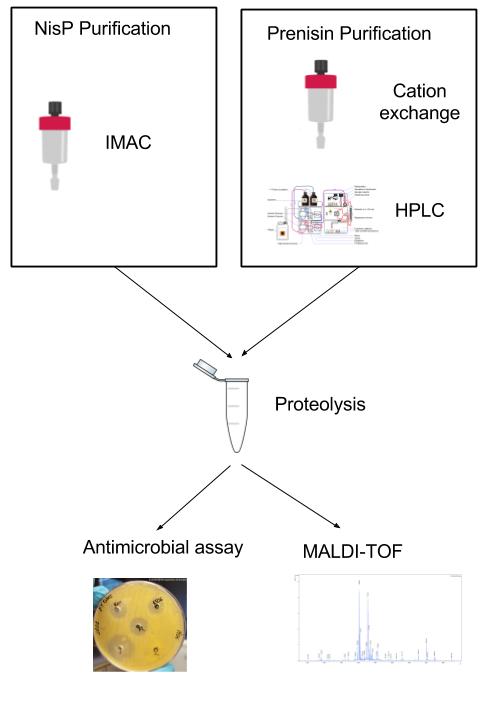

Supplement: Supplementary Figure 1 — Schematic view of a possible workflow for the biotechnological application of NisP using nisin as an example. [file Image1.JPEG]
