# Supplementary material for: Specificity and Application of the Lantibiotic Protease NisP
Source: Front Microbiol. 2018 Feb 9;9:160. doi: 10.3389/fmicb.2018.00160 (PMC5812297; doi:10.3389/fmicb.2018.00160)
Supplement: Supplementary file 2 [file Table1.docx]

Supplementary Material

**Specificity and Application of the Lantibiotic Protease NisP**

Manuel Montalbán-López^1,2#^, Jingjing Deng^1#^, Auke J. van Heel^1^, Oscar P. Kuipers^1^*

*** Correspondence:** Corresponding Author: Oscar P. Kuipers o.p.kuipers@rug.nl

Supplementary table 1: Primers used in this study

| Name | Sequence | Purpose |
| --- | --- | --- |
| nisPbsphfwd | CGT GAG TCA TGA AAA AAA TAC TAG GTT TCC | Amplification of NisP |
| nisP8KXbarev | TCG TGT CTA GAT TAT TTC TTT TTC TTT TTC TTT TTC TTT TGA CTT CGT ACA GAA ACA GC | Amplification of NisP without sortase sequence and 8-Lys |
| nisP8HXbarev | TCG TGT CTA GAT TAA TGG TGA TGG TGA TGG TGA TGG TGT TGA CTT CGT ACA GAA ACA GC | Amplification of NisP without sortase sequence and 8-His |
| nisP8KSacIr | TCGT GGA GCT CT TAC TTT TTC TTT TTC TTT TTC TTT TTA TCT GTA TCT AAG CTA AAA GC | Amplification of truncated NisP without cell wall helix and with 8-Lys |
| solnisP8HSacIr | TCGT GGA GCT CT TAA TGG TGA TGG TGA TGG TGA TGG TGA TCT GTA TCT AAG CTA AAA GC | Amplification of truncated NisP without cell wall helix and with 8-His |
| solnisPcontrol | TCGT GGA GCT CT TAA TCT GTA TCT AAG CTA AAA GC | Amplification of truncated NisP without cell wall helix |
| nisPrev2 | GAC AAT ATC ACT TGG ATT TCC | sequencing |
| nisPrev2 | GTT GTG CCA GCA GGA GC | sequencing |
| nisP8K/H | GA CTT CGT ACA GAA ACA GC | sequencing |
| pNZE3Emf | CAA TTC CTT AAA ACA TGC AGG | sequencing |
| pNZE3revMML | CAA TCA AAG CAA CAC GTG C | sequencing |
| C-lessH6-less | TC TAG AAG CTT ATT TGC TTA CGT GAA TAC TAG CAT GAG C | Removal of the his-tag in nisin C-less |
| NisPC7A-rev | GCT AGC GAA ATA CTT GTA ATA CGA AGT GAA ACA CCT GAA TC | Construction of nisin C7A-VSLR |
| NisPC7A-fwd | GTA TTA CAA GTA TTT CGC TAG CTA CAC CCG GTT GTA AAA CAG G | Construction of nisin C7A |
| nisVSLRfwd | CTACGCATTACAAGTATTTCGC | Creation of the VSLR cleavage sequence |
| nisVSLRrev | ACT TAC ACC TGA ATC TTT CTT CG | Creation of the VSLR cleavage sequence |
| P-for | AGTATTTCGCTATGTACACCCGGTTG | Mutation of residues I1 or T2 of nisin |
| P-IK-Rev | TTTAATACGAAGTGAAACACCTGAATCTTTCTTCGAAAC | Mutation T2K |
| P-KT-Rev | AGTTTTACGAAGTGAAACACCTGAATC | Mutation I1K |
| P-WT-Rev | AGTCCAACGAAGTGAAACACCTGAATC | Mutation I1W |
| P-DT-Rev | AGTATCACGAAGTGAAACACCTGAATC | Mutation I1D |
| P-IV-Rev | CCTGACTTTTTCTTACGAAGTGAAACACCTGAATC | Mutation T2V |
| NisPC7A-ASPR-rev | TAGCGAAATACTTGTAATACGTGGTGATGCACCTGAATC | Construction of nisin C7A-ASPR |
| ringAdel-fwd | TGTTGCAATTGCGCTAGCTACACCCGGTTGTAAAACAGG | Mutation of dehydratable residues N-terminal to T8 |
| RingAless-ASPR-rev | TAG CGC AAT TGC AAC AAT GCG TGG TGA TGC ACC TGA ATC | Mutation of dehydratable residues N-terminal to T8 |
| RingAless-VSLR-rev | TAG CGC AAT TGC AAC AAT ACG TAA TGA AAC ACC TGA ATC | Mutation of dehydratable residues N-terminal to T8 |
